# Depletion of gut microbiota alleviates proteinuria in puromycin aminonucleoside-induced nephrosis in rats

**DOI:** 10.1038/s41390-025-04668-9

**Published:** 2025-12-15

**Authors:** Saikhanchimeg Myagmankhuu, Shoji Tsuji, Shohei Akagawa, Jiro Kino, Yuko Akagawa, Sohsaku Yamanouchi, Kazunari Kaneko

**Affiliations:** 1 Department of Pediatrics, Kansai Medical University, Hirakata, Osaka Japan; 2 Osaka Asahi Children’s Hospital, Osaka, Japan

## Abstract

**Background:**

The gut–kidney axis has been implicated in chronic kidney disease, however its role in minimal change nephrotic syndrome (MCNS) is poorly understood. We investigated the impact of gut microbiota on proteinuria in MCNS.

**Methods:**

A puromycin aminonucleoside (PAN)-induced rat model of MCNS was used. Rats received a cocktail of antibiotics, PBS (control), or antibiotics plus indoxyl sulfate (IS). To assess causality, fecal microbiota transplantation (FMT) was performed in additional PAN rats. Urinary protein, kidney histology, urinary IS, 8-hydroxy-2′-deoxyguanosine (8-OHdG), and gut microbiota composition were evaluated.

**Results:**

On day 8 after PAN injection, antibiotic-treated rats exhibited markedly reduced proteinuria (1.4 g/gCre) compared with controls (16.8 g/gCre, *p* = 0.014), whereas IS-treated rats developed severe proteinuria (117.3 g/gCre). Electron microscopy revealed podocyte foot process effacement in control and IS-treated rats but not in antibiotic-treated rats. Antibiotic-treatment decreased indole-producing bacteria, lowered urinary IS, and reduced 8-OHdG levels, indicating attenuation of oxidative stress. Importantly, FMT abolished the protective effect of antibiotics, re-emerging proteinuria.

**Conclusion:**

Depletion of the gut microbiota by antibiotic treatment in a rat MCNS model alleviated proteinuria, which was reversed by FMT. This causally implicates gut microbiota, particularly indole-producing bacteria that generate toxins including IS, as a key therapeutic target for MCNS.

**Impact:**

- This study demonstrated that depleting the gut microbiota with antibiotics reduced proteinuria in a rat model of minimal change nephrotic syndrome, suggesting that harmful gut bacteria play a critical role in this disease.
- This research also identified indoxyl sulfate as a key uremic toxin produced by gut bacteria that worsens proteinuria and kidney damage, highlighting its role in disease progression.
- These findings could lead to novel treatments that target gut microbiota, including antibiotics or activated charcoal adsorbents that reduce proteinuria in minimal change nephrotic syndrome, and potentially minimize steroid use.

## Introduction

Nephrotic syndrome (NS) is a disease characterized by massive proteinuria. Large amounts of serum protein leak into the urine, causing hypoalbuminemia, edema, hypercoagulable states, increased susceptibility to infection, and impaired fluid balance regulation. Approximately 90% of childhood NS is classified as idiopathic nephrotic syndrome (INS), approximately 10% as secondary, and approximately 1% as congenital. In addition, childhood INS can be divided into two major categories: minimal change nephrotic syndrome (MCNS), which accounts for 90% of cases, and focal segmental glomerulosclerosis, which accounts for 10% of cases.^1,2^

Although the pathogenesis of massive proteinuria in MCNS is not yet fully understood, several recent reports, including ours, have investigated the relationship between MCNS and a disturbance of the gut microbiota, termed gut dysbiosis.^3–9^ These studies reported that gut dysbiosis was characterized by a smaller proportion of beneficial bacteria, such as short-chain fatty acid (SCFA)-producing bacteria, and an increased abundance of harmful bacteria in patients with INS.

However, it is unclear whether a decrease in beneficial bacteria or an increase in harmful bacteria would have a more critical role in the appearance of proteinuria in MCNS. Therefore, we investigated the effect of the gut microbiota on proteinuria in MCNS using rats with puromycin aminonucleoside (PAN)-induced nephropathy (PAN rats).

## Materials and methods

### Animals and study protocol

The experimental protocols were approved by the Animal Research Committee of Kansai Medical University (no. 24-080). Female Wistar rats (4 weeks old) were purchased from Shimizu Laboratory Supplies Co., Ltd. (Kyoto, Japan).

**Fig. 1:**
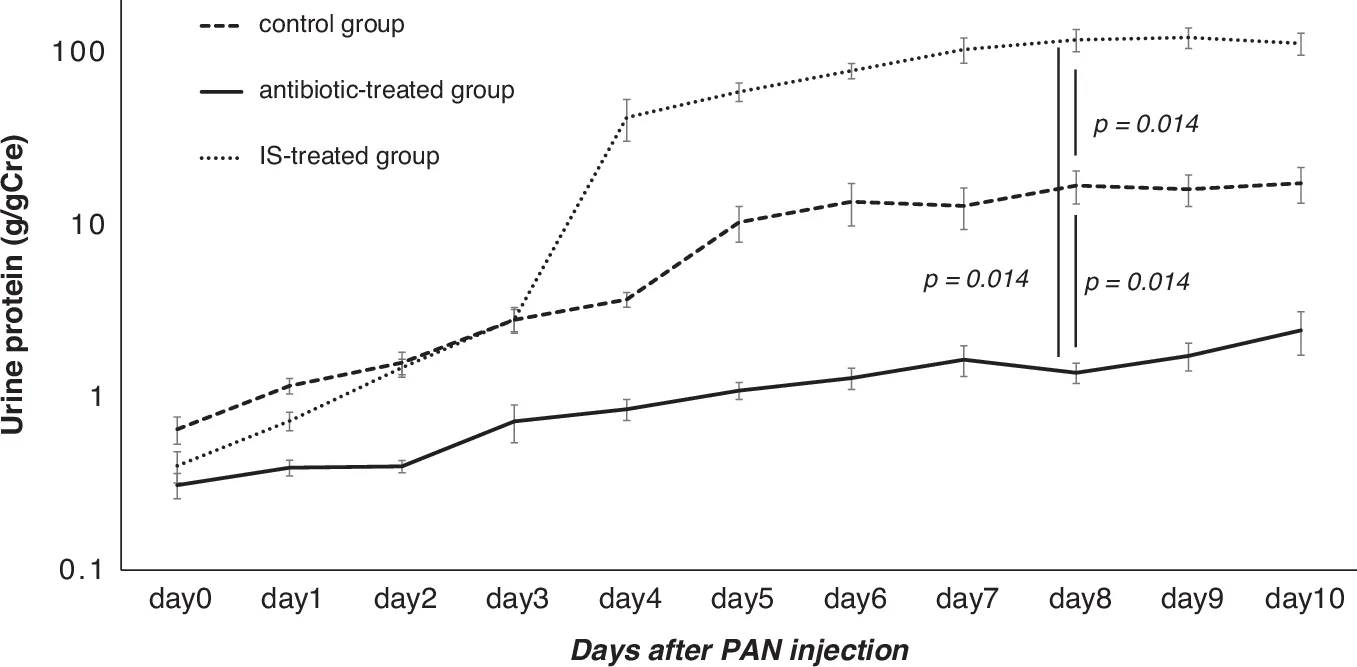
Proteinuria in Wistar rats after the subcutaneous injection of PAN. The subcutaneous injection of PAN to Wistar rats (*n* = 6) after the oral administration of PBS for 10 days (control group) induced proteinuria, the amount of which gradually increased until day 8 (broken line). By contrast, in the group that received oral antibiotics for 10 days beforehand (antibiotic-treated group, *n* = 6), proteinuria was significantly reduced compared with the control group for all days after day 1 (solid line). Conversely, in the IS-treated group (*n* = 6) that received the same antibiotics as the antibiotic-treated group for 10 days, followed by a single simultaneous administration of PAN and IS, the amount of urinary protein was significantly higher than in the control and the antibiotic-treated groups for all days (dotted line). There were significant differences in the amount of proteinuria among the three groups for all days after day 1 as assessed by the Kruskal-Wallis test. Multiple comparisons of the three groups by the Steel-Dwass test following the Kruskal-Wallis test are shown in Table 1. On day 8, at the peak of proteinuria, the urinary protein levels were significantly lower in the antibiotic-treated than in the control group (antibiotic-treated group vs the control group: 1.4 vs 16.8 g/gCre, *p* = 0.014). In comparison, it was significantly higher in the IS-treated group than in the control group and antibiotic-treated group (IS-treated group vs control group vs antibiotic-treated: 117.3 vs 16.8 vs 1.4 g/gCre, p < 0.01). Concurrently, urine creatinine was also measured by an enzymatic method, and the urine protein quantity was corrected for creatinine. PAN puromycin aminonucleoside, PBS phosphate-buffered saline.

**Fig. 2:**
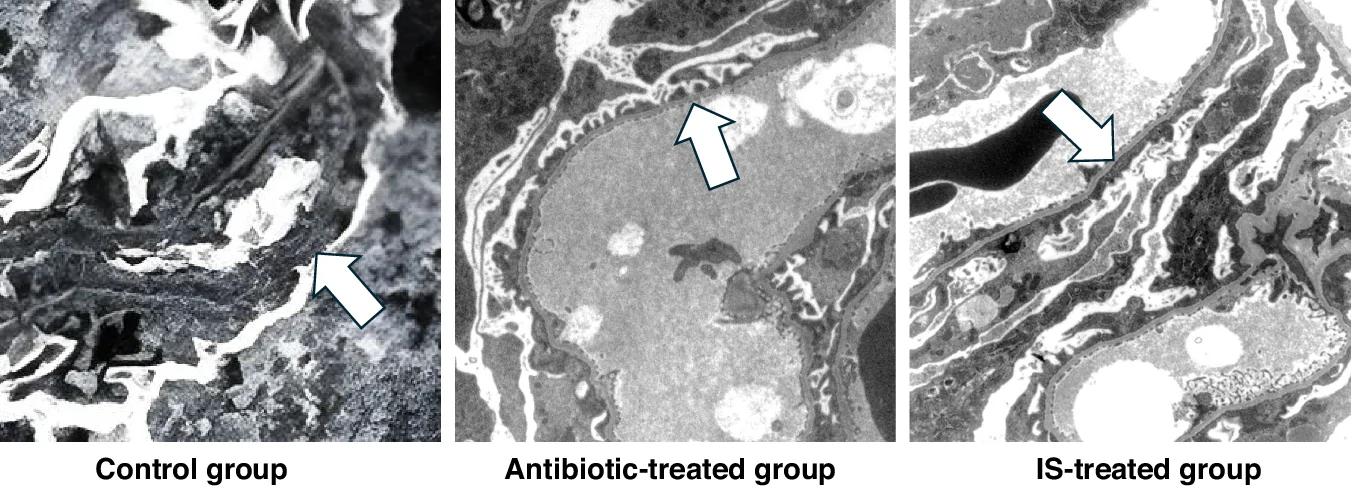
Electron microscopic analysis of the kidneys of rats injected subcutaneously with PAN. Representative electron microscopic images of the renal glomeruli of rats 10 days after PAN subcutaneous injection are shown. Electron microscopic analysis revealed the flattening of podocytes and effacement of foot processes in the renal glomeruli of the PBS administration group (control group: **a** arrow, magnification ×12,000). In the renal glomeruli of PAN subcutaneous injection rats pretreated with antibiotics, no effacement of foot processes was observed (antibiotic-treated group; **b** arrow, magnification ×12,000). In the IS-treated group, in which IS was administered once simultaneously with PAN 10 days after antibiotic administration, the effacement of podocyte foot processes was observed (**c** arrow, magnification ×12,000). IS indoxyl sulfate, PAN puromycin aminonucleoside, PBS phosphate-buffered saline.

**Fig. 3:**
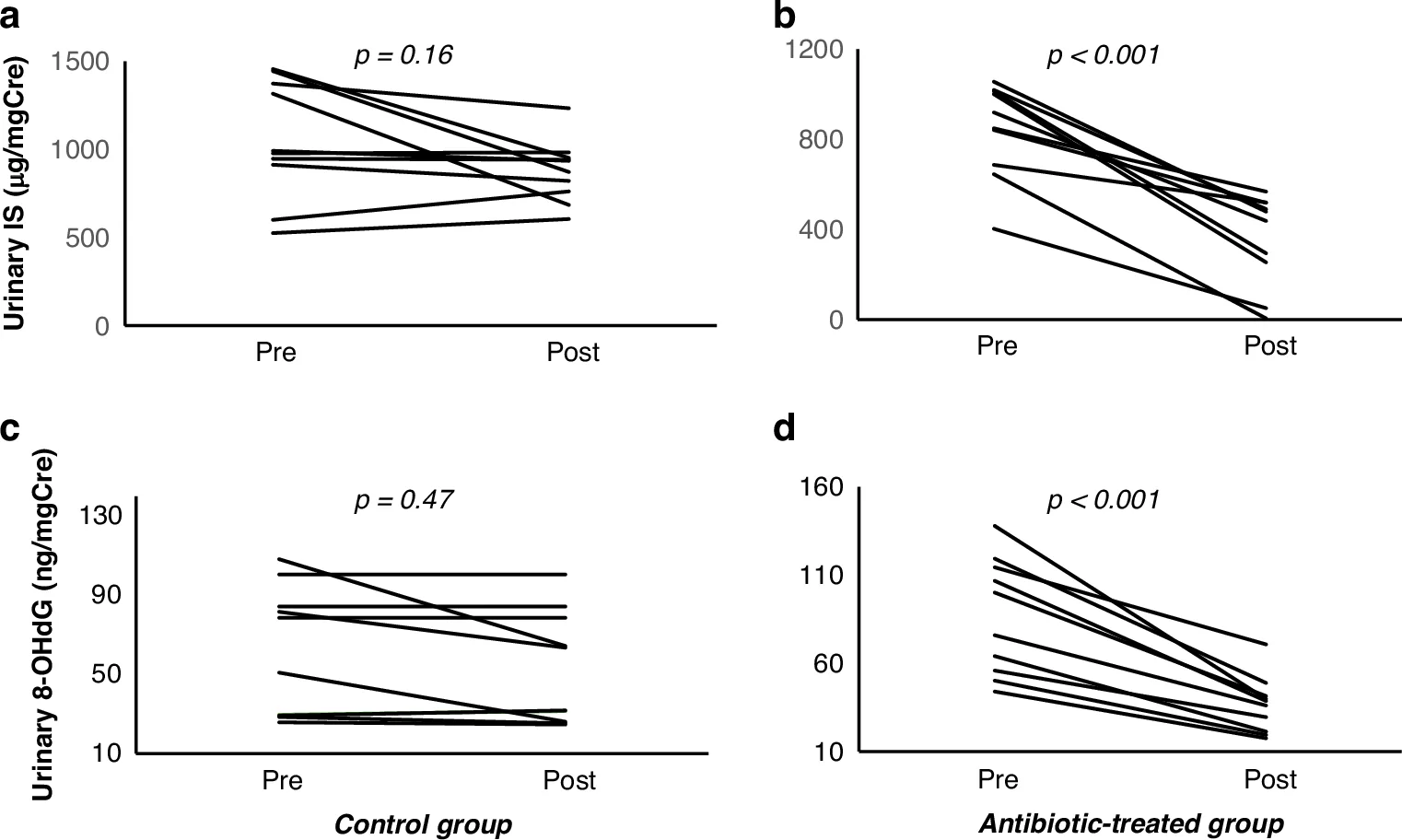
Changes in urinary IS and 8-OHdG after the administration of antibiotics to PAN rats. **a**, **c** Changes in urinary IS and 8-OHdG in the control group. **b**, **d** Changes in urinary IS and 8-OHdG in the antibiotic-treated group. These measurements were performed on a different group of rats from the primary groups to assess proteinuria (*n* = 10 per group). There was no significant change in urinary IS levels in the control group before PBS administration and 10 days after PBS administration (*p* = 0.16, **a**). By contrast, in the antibiotic-treated group, the mean urinary IS levels were significantly decreased (*p* < 0.001, **b**). Similarly, changes in the mean urinary levels of 8-OHdG, a marker of oxidative stress, before PBS administration and 10 days after PBS administration did not reach statistical significance in the control group (*p* = 0.47, **c**). However, in the antibiotic-treated group, the mean urinary 8-OHdG levels were significantly decreased 10 days after antibiotic administration (*p* < 0.001, **d**). IS Indoxyl sulfate, 8-OHdG 8-hydroxy-2′-deoxyguanosine, PAN puromycin aminonucleoside, PBS phosphate-buffered saline, Cr creatinine.

**Fig. 4:**
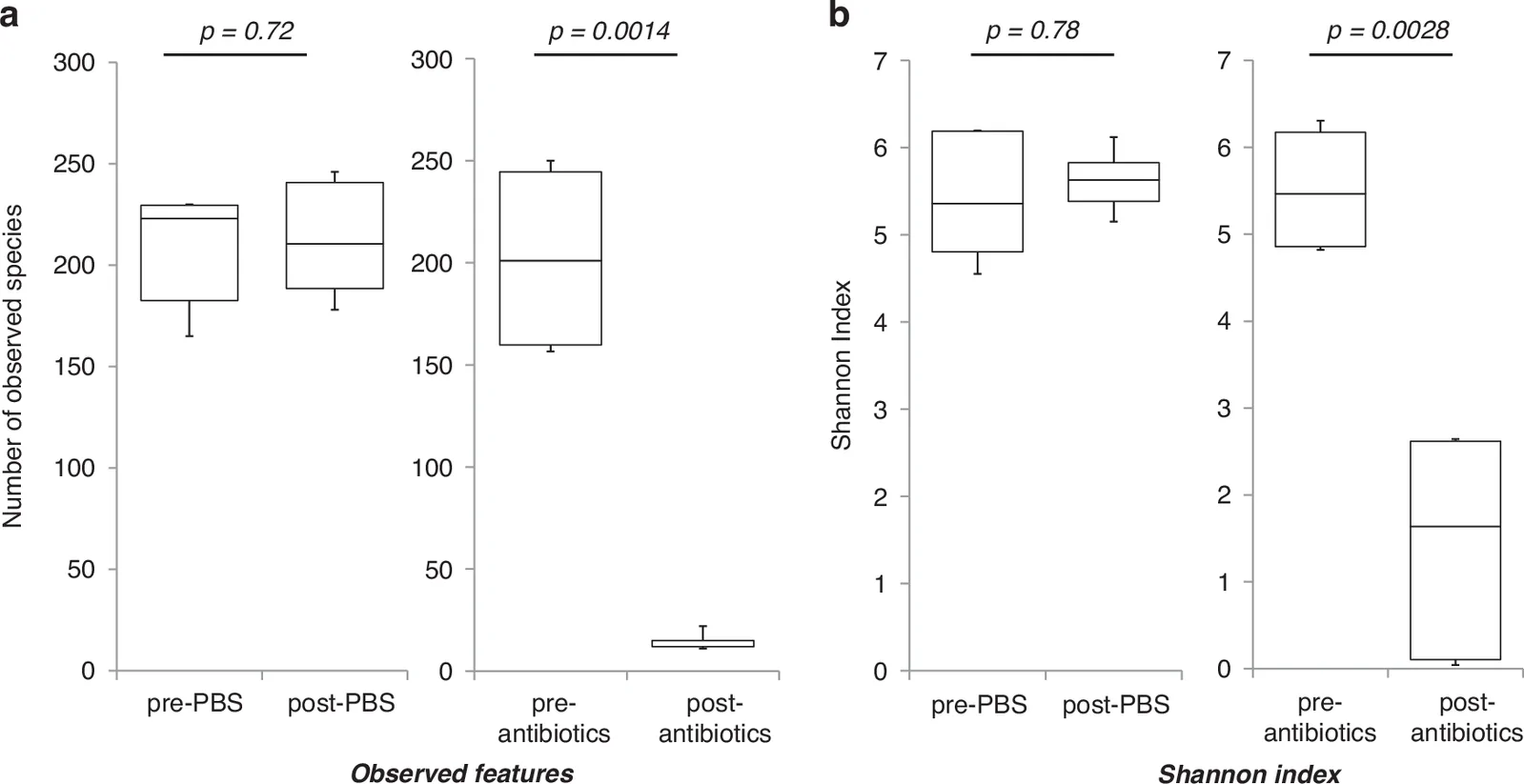
Changes in the alpha-diversity of the gut microbiota before and after the administration of antibiotics or PBS to PAN rats. **a** Observed features of the gut microbiota. **b** The Shannon index of the gut microbiota. Observed features of the gut microbiota indicating the number of observed species did not change significantly before and after PBS administration (*p* = 0.72), but were significantly decreased after 10 days of antibiotic administration (*p* = 0.0014). Similarly, Shannon index, which indicates the abundance and homogeneity of bacterial species, showed no significant change before and after PBS administration (*p* = 0.78), but was significantly lower after 10 days of antibiotic administration (*p* = 0.0028). PBS phosphate-buffered saline, PAN puromycin aminonucleoside.

**Fig. 5:**
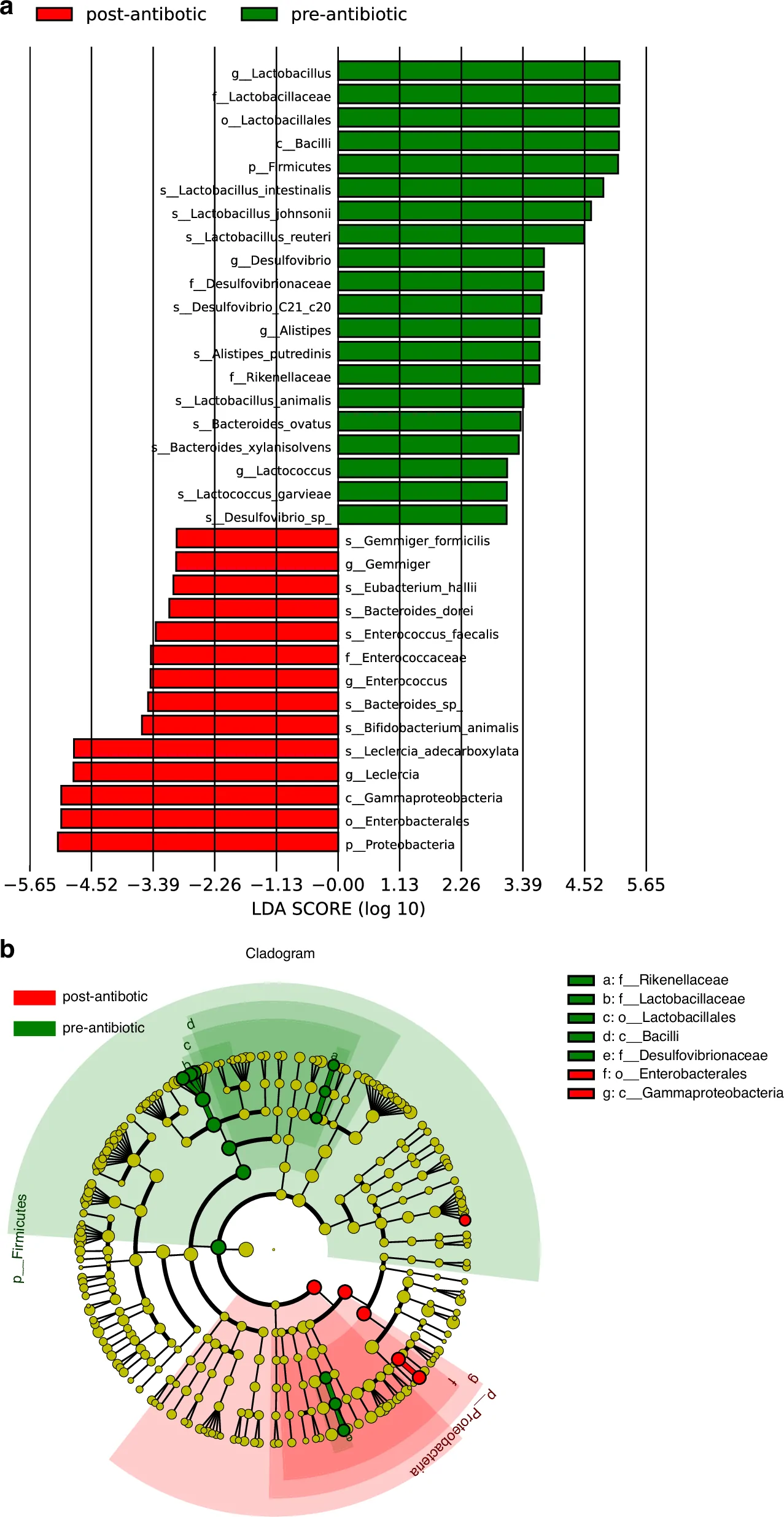
Compositional changes in the gut microbiota before and after antibiotic administration to PAN rats. **a** Linear discriminant analysis (LDA) histogram showing differentially abundant bacterial taxa. **b** Cladogram representing the phylogenetic distribution of the gut microbiota. The figure shows an LDA histogram, including species (upper panel) and cladogram (continuous circles, representing phylogenetic levels [phylum, order, family, and genus]; lower panel) based on LEfSe analysis. The LEfSe results show that the genera *Alistipes* and *Desulfovibrio*, and *Bacteroides spp*. involved in indoxyl sulfate production are reduced after antibiotic treatment. LDA linear discriminant analysis, LEfSe Linear discriminant analysis Effect Size, PAN puromycin aminonucleoside.

**Fig. 6:**
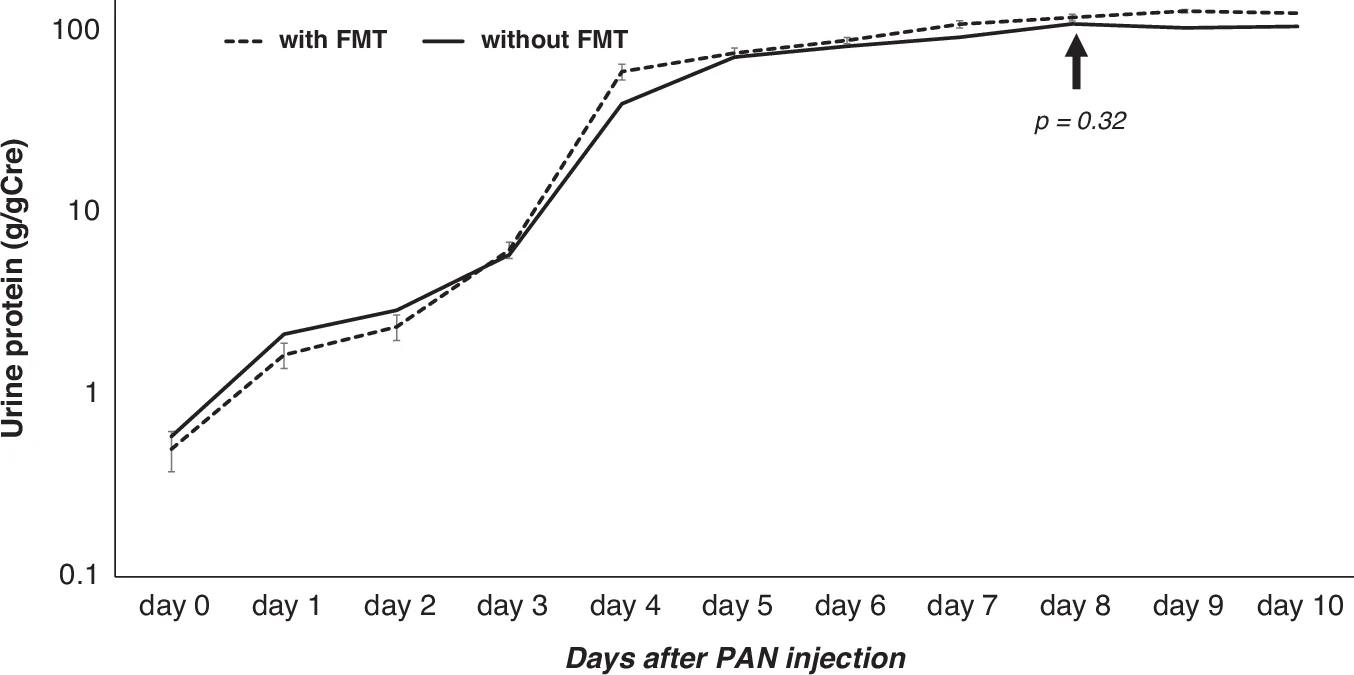
FMT abolishes the anti-proteinuric effect of antibiotics in PAN rats. The line chart displays the changes in urinary protein levels (g/gCre) for 10 days after PAN injection. The comparison was made between the control group (“without-FMT” PBS + PAN, *n* = 6: solid line) and the group that received FMT after antibiotic treatment (“with-FMT” antibiotic-treated + FMT + PAN, *n* = 7: broken line). There was no statistically significant difference in the amount of proteinuria between the “with-FMT group” and the “without-FMT group” on day 8 post-PAN injection, at the peak of proteinuria (120.3 g/gCre and 110.8 g/gCre, respectively, *p* = 0.32). FMT fecal microbiota transplantation, PAN puromycin aminonucleoside, PBS phosphate-buffered saline administration, all rats received a single subcutaneous injection of PAN (50 mg/kg) on day 0 and were monitored for 10 days. FMT fecal microbiota transplantation, PAN puromycin aminonucleoside, PBS phosphate-buffered saline.

The study protocol involved three separate groups of animals. For the primary experiment assessing proteinuria and renal histology (Figs. 1, 2, and S1), PAN nephropathy was induced in six rats per experimental group. For the specific measurement of urinary markers such as indoxyl sulfate (IS) or 8-hydroxy-2′-deoxyguanosine (8-OHdG), and microbial analysis (Figs. 3–5), ten rats each for the control and antibiotic-treated groups were provided. For an experiment of fecal microbiota transplantation (FMT) (Fig. 6 and S2), two sets of rats were used. Seven rats with the ”with-FMT group” were treated with the antibiotics followed by the FMT, while six rats with the “without-FMT group” served as the control only received phosphate-buffered saline (PBS) instead of antibiotics and the FMT.

PAN nephropathy was induced by a single subcutaneous injection of PAN (Sigma, St. Louis, MO) dissolved in PBS at a dose of 50 mg/kg body weight. The PAN-induced nephropathy rat model was utilized because it is a well-established experimental model that faithfully reproduces the key pathological features of human MCNS, including podocyte foot process effacement and massive proteinuria.^10,11^

### Effect of antibiotics and IS on proteinuria in PAN rats

As shown in Fig. S1, rats in the antibiotic-treated group were administered amoxicillin, cefotaxime, vancomycin, and metronidazole, with the types and dosages of antibiotics referenced from previous reports.^12,13^ Specifically, amoxicillin (150 mg/kg/day), cefotaxime (20 mg/kg/day), vancomycin (20 mg/kg/day), and metronidazole (20 mg/kg/day) were administered orally to rats daily for 10 days before the administration of PAN. The administration of this combination of antibiotics was reported to deplete the gut microbiota in rats.^14^ Ten days after the oral administration of the four antibiotics listed above, rats in the IS-treated group received a single intraperitoneal injection of IS (800 mg/kg, Sigma), a uremic toxin produced in the liver following the absorption of indole from the intestine, concurrent with the administration of PAN. This dosage was based on the work of Ichii et al., who demonstrated the IS-induced podocyte injury in a rodent model.^15^ In addition, a large dose was used to achieve transiently elevated plasma levels in animals with normal renal function because of the rapid glomerular and tubular excretion of IS. A control group of rats received 2 mL/day of PBS alone orally every day for 10 days before the administration of PAN. Spot urine samples were collected daily from all rats throughout the experimental period of 10 days after PAN administration. Urinary protein and creatinine levels were measured using pyrogallol red and enzymatic methods, respectively, and levels of urinary protein were expressed as ratios to the urinary creatinine level to account for variations in urinary dilution.

### Transmission electron microscopy

On day 10 after PAN administration of the above-mentioned protocol, rats were anesthetized by isoflurane inhalation and sacrificed. For ultrastructural analysis, the renal cortex was dissected after whole-body perfusion with PBS. The tissues were immediately fixed with 2% glutaraldehyde overnight, postfixed with 1% osmium tetroxide, dehydrated in a graded series of acetone dilutions, and embedded in Epon-Araldite. Ultrathin sections cut at a thickness of 0.08 μm were stained with uranyl acetate and lead citrate, and examined using a Hitachi H-7100 transmission electron microscope (Tokyo, Japan).

### Changes in urinary IS and 8-OHdG after the administration of antibiotics

Using a separate set of rats from those used for urine protein measurements, the urinary levels of IS and 8-OHdG, a marker of oxidative stress, were also determined in ten rats in the control and antibiotic-treated groups before and after the administration of PBS or antibiotics using high-performance liquid chromatography (measurements performed at FUSHIMI Pharmaceutical Co., Ltd., Kagawa, Japan) and the ICR-001 urinary oxidative stress marker measurement system (Techno Medica Co., Ltd., Yokohama, Japan), respectively. To account for variations in urinary dilution, the levels of urinary IS and 8-OHdG were expressed as ratios to the urinary creatinine level.

### Fecal sample collection and 16S rRNA sequencing

To analyze the gut microbiota, rats provided for measurements of urinary IS and 8-OHdG were also used. Fecal samples were collected from these rats through natural defecation before and after the administration of PBS or antibiotics in the control and antibiotic-treated groups. The collected samples were stored at −80 °C until analysis. Within 1 week of collection, DNA was extracted from the fecal samples using the NucleoSpin DNA Stool Kit (MACHEREY-NAGEL, Düren, Germany). 16S rRNA gene sequencing was conducted by Macrogen Japan Corp. (Tokyo, Japan). Sequencing libraries were prepared according to the Illumina 16S Metagenomic Sequencing Library Preparation protocol, targeting the V3 and V4 hypervariable regions for amplification. Two nanograms of genomic DNA extracted from fecal samples were combined with 5× reaction buffer, 1 mM deoxynucleotide triphosphate mix, 500 nM universal forward and reverse polymerase chain reaction (PCR) primers, and Herculase II Fusion DNA Polymerase (Agilent Technologies, Santa Clara, CA) for PCR amplification. The cycling conditions for the initial PCR were as follows: 95 °C for 3 min for initial denaturation, followed by 25 cycles of 95 °C for 30 s (denaturation), 55 °C for 30 s (annealing), and 72 °C for 30 s (extension), with a final extension at 72 °C for 5 min. For the initial amplification, the universal primer pair with Illumina adapter overhang sequences were as follows: V3-forward: 5′-tcgtcgcgcagcgtcagatggtagtataagacagcctacgggncwgcag-3′; V4-reverse: 5′-gtctctcgtgcgctctcgagatgatagatagagacaggactachvgggtactaatcc-3′.

The 1st round PCR products were purified with AMPure beads (Agencourt Bioscience, Beverly, MA). After purification, 2 µL of the 1st round PCR product was amplified in a 2nd round of PCR to construct the final library, including indexing with Nextera XT Indexed Primers. The cycling conditions for the 2nd round PCR were the same as for the 1st round PCR, except for a reduction in the number of cycles for the amplification step to 10 cycles. The PCR products were purified with AMPure beads. The final purified products were quantified by quantitative PCR according to the qPCR Quantification Protocol Guide (KAPA Library Quantification Kit for Illumina Sequencing Platforms) and qualified using a TapeStation D1000 ScreenTape (Agilent Technologies, Waldbronn, Germany).

Sequencing reads were imported into the Quantitative Insights Into Microbial Ecology version 2 (QIIME2) pipeline (version 2021.11)^16^ for bacterial identification and diversity analysis. Alpha diversity was calculated using the Shannon index and Observed features. The linear discriminant analysis effect size (LEfSe) was used to analyze differences in taxa abundance.

### FMT in rats

To confirm the causal relationship between gut microbiota and proteinuria in PAN rats, we additionally performed an FMT experiment. The protocol was adapted from previously published methods.^8,17^ Briefly, fecal samples for transplantation were collected from untreated, healthy Wistar rats into sterile tubes. The samples were suspended in sterile PBS at a ratio of 100 mg of feces per 1 mL of PBS and then homogenized by vortexing for 5 min. The resulting fecal slurry was filtered through sterile gauze, and then the filtrate was centrifuged at 800 × *g* for 3 min to pellet large particulate matter. The supernatant was collected carefully, and sterile glycerol was added as a cryoprotectant to a final concentration of 10%. Aliquots of the prepared fecal transplant material were stored at −80 °C until use.

As shown in Fig. S2, rats were divided into two groups for the FMT experiment. The “with-FMT group (*n* = 7)” was treated with the antibiotic cocktail (amoxicillin, cefotaxime, vancomycin, and metronidazole) for 10 days to deplete the native gut microbiota. Following the antibiotic course, these rats received 200 µL of the thawed fecal slurry daily via oral gavage for 7 consecutive days. The “without-FMT group (*n* = 6)” served as the control and received the daily oral administration of PBS (2 mL/day) instead of antibiotics for the first 10 days, followed by an equal volume of PBS via oral gavage for the next 7 days. Upon completion of the treatment period for both groups, nephropathy was induced by a single injection of PAN (50 mg/kg). Thereafter, daily urinary protein measurements using spot urine samples collected from all rats throughout the experimental period of 10 days after PAN administrations were performed.

### Statistical analysis

All values are expressed as the mean ± standard error of the mean. Pairwise comparisons between two groups were performed using the Wilcoxon signed-rank test. Comparisons between three groups were performed using the Kruskal–Wallis test. The Steel-Dwass test was used for multiple comparisons of three groups following the Kruskal–Wallis test. Statistical significance was defined as *p* < 0.05. Analyses were performed using JMP Pro 17 statistical software (JMP; Cary, NC).

## Results

### The oral administration of a combination of antibiotics reduces PAN-induced proteinuria in rats

Figure 1 shows the changes in urinary protein levels after PAN injection in three groups of rats consisting of a control group (broken line), an antibiotic-treated group (solid line), and an IS-treated group (dotted line). There were significant differences in proteinuria among the three groups for all days from day 1, as determined by the Kruskal–Wallis test. The control group gradually developed massive proteinuria after PAN injection, peaking on day 8. By contrast, the antibiotic-treated group showed significantly lower proteinuria even after PAN injection. Meanwhile, the IS-treated group demonstrated the highest level of proteinuria among the three groups. Multiple comparisons of the three groups by the Steel–Dwass test following the Kruskal–Wallis test are shown in Table 1. On day 8, at the peak of proteinuria, the urinary protein levels were significantly lower in the antibiotic-treated group than in the control group (antibiotic-treated group vs the control group: 1.4 vs 16.8 g/gCre, *p* = 0.0141). In comparison, it was significantly higher in the IS-treated group than in the control group and the antibiotic-treated group (IS-treated group vs control group vs antibiotic-treated group: 117.3 vs 16.8 vs 1.4 g/gCre, *p* < 0.01).

**Table 1:** Multiple comparisons of proteinuria in each group by the Kruskal–Wallis test followed by Steel–Dwass analysis.

		Difference in mean scores	95% CI	*P* value
Day 1	Cont vs. ATBs.	5.83	0.24–1.21	0.014
	IS vs. ATBs	4.50	−0.095–0.74	0.078
	Cont vs. IS	3.83	−0.077 to 1.01	0.16
Day 2	Cont vs. ATBs.	5.83	0.63–2.19	0.014
	IS vs. ATBs	5.83	0.45–1.51	0.014
	Cont vs. IS	0.50	−0.76–1.57	0.97
Day3	Cont vs. ATBs.	5.50	0.26–3.31	0.022
	IS vs. ATBs	5.50	0.45–3.72	0.022
	Cont vs. IS	−0.17	−2.77–2.24	0.10
Day 4	Cont vs. ATBs.	5.83	1.45–4.22	0.014
	IS vs. ATBs	5.83	3.63–70.87	0.014
	Cont vs. IS	−5.50	−44.06 to −68.48	0.022
Day 5	Cont vs. ATBs.	5.83	2.04–19.79	0.014
	IS vs. ATBs	5.83	26.52–72.72	0.014
	Cont vs. IS	−5.83	−68.57 to −15.11	0.014
Day 6	Cont vs. ATBs.	5.83	2.20–29.13	0.014
	IS vs. ATBs	5.83	44.41–91.20	0.014
	Cont vs. IS	−5.83	−87.92 to −30.66	0.014
Day 7	Cont vs. ATBs.	5.83	2.23–26.02	0.014
	IS vs. ATBs	5.83	21.68–146.46	0.078
	Cont vs. IS	−5.50	−139.65 to −5.32	0.16
Day 8	Cont vs. ATBs.	5.83	3.53–26.45	0.014
	IS vs. ATBs	5.83	34.80–146.47	0.014
	Cont vs. IS	−5.83	−140.62 to −9.45	0.014
Day 9	Cont vs. ATBs.	5.83	3.62–25.72	0.014
	IS vs. ATBs	5.83	39.17–149.01	0.014
	Cont vs. IS	−5.83	−140.23 to −16.76	0.014
Day 10	Cont vs. ATBs.	5.83	4.49–32.08	0.014
	IS vs. ATBs	5.83	30.27–140.15	0.014
	Cont vs. IS	−5.50	−129.51 to −7.73	0.022

*Cont* control group, *ATBs* antibiotic-treated group, *IS* IS-treated group.

### Electron microscopic findings of rat glomerular podocytes exhibit no effacement of foot processes in the antibiotic-treated PAN rats

Evaluation of rat glomerular podocytes by electron microscopy after PAN administration showed that the control group exhibited the effacement of foot processes related to podocyte injury caused by PAN (Fig. 2a, arrow). However, the antibiotic-treated group maintained the structure of podocyte foot processes, even after PAN administration (Fig. 2b arrow). By contrast, the IS-treated group, which received a single dose of IS simultaneously with PAN after 10 days of antibiotic administration, showed the effacement of podocyte foot processes similar to that in the control group (Fig. 2c arrow).

### Antibiotic treatment decreases urinary IS and 8-OHdG in PAN rats

Urinary levels of IS, a uremic toxin completely derived from gut microbiota and 8-OHdG, a marker of oxidative stress, were also assessed in ten rats before and after the administration of PBS or antibiotics in the control and antibiotic-treated groups, respectively. Mean urinary IS levels corrected by creatinine in the control group were 1054.2 ± 106.0 µg/mgCr before PBS administration and 878.7 ± 55.7 µg/mgCr 10 days after PBS administration, which did not reach statistical significance (*p* = 0.16, Fig. 3a). By contrast, in the antibiotic-treated group, the mean urinary IS levels corrected by creatinine were significantly decreased (842.8 ± 66.0 µg/mgCr before antibiotic administration and 361.8 ± 63.9 µg/mgCr 10 days after antibiotic administration; *p* < 0.001, Fig. 3b).

Similarly, mean urinary 8-OHdG levels corrected by creatinine in the control group were 60.5 ± 9.8 ng/mgCre before PBS administration and 50.0 ± 7.0 ng/mgCre 10 days after PBS administration, which did not reach statistical significance (*p* = 0.47, Fig. 3c). However, in the antibiotic-treated group, the mean urinary 8-OHdG levels corrected by creatinine were significantly decreased (86.9 ± 9.4 ng/mgCre before antibiotic administration and 36.3 ± 4.6 ng/mgCre 10 days after antibiotic administration; *p* < 0.001, Fig. 3d).

### Antibiotic treatment drastically changes the gut microbiota in PAN rats

Figure 4 demonstrates the results of the alpha-diversity analysis of the gut microbiota before and after the administration of PBS or antibiotics in the control and antibiotic-treated groups, respectively. Observed features, which indicate the number of observed species, were not significantly changed before and after PBS administration (Fig. 4a, *p* = 0.72) in the control group but showed a statistically significant decrease 10 days after antibiotic administration compared with that before antibiotic administration (Fig. 4a, *p* = 0.0014) in the antibiotic-treated group. Similarly, Shannon index, which indicates species richness and evenness, also showed no significant change before and after PBS administration (Fig. 4b, *p* = 0.78); however, it showed a statistically significant decrease 10 days after antibiotic administration compared with that before administration (Fig. 4b, *p* = 0.0028).

Figure 5 shows compositional changes in the gut microbiota before and after antibiotic administration, assessed by a cladogram based on LEfSe (concentric circles represent phylogenetic levels: phylum, class, order, family, and genus) and a linear discriminant analysis (LDA) histogram including species in the antibiotic-treated group. Consistent with the observed decrease in urinary IS, LEfSe analysis showed that several genera known to produce indole—the precursor to IS—were significantly decreased after antibiotic administration. These included *Alistipes*, *Bacteroides*, and *Desulfovibrio*, which possess the enzyme tryptophanase, which metabolizes tryptophan into indole.^18,19^

### FMT abolishes the anti-proteinuric effect of antibiotics in PAN rats

To definitively establish a causal role for the gut microbiota in the pathogenesis of PAN-induced proteinuria, we compared PAN-induced proteinuria in antibiotic-pretreated rats that subsequently received an FMT from healthy donors (“with-FMT group”) with a control group that received PBS instead of antibiotics and did not receive an FMT (“without-FMT group”). Figure 6 shows the “with-FMT group” (broken line) developed severe proteinuria following PAN administration, comparable to the “without-FMT group” (solid line). This finding demonstrates that the protective effect of the antibiotic treatment in PAN rats was completely abolished by the FMT. Specifically, on day 8 post-PAN injection, at the peak of proteinuria, the proteinuria level in the “with-FMT group” (120.3 g/gCre) was comparable with that of the “without-FMT group” (110.8 g/gCre), with no statistically significant difference (*p* = 0.32). This result demonstrates that restoring the gut microbiota in antibiotic-treated rats by FMT was sufficient to reverse the anti-proteinuric effect of gut microbiota depletion, providing direct evidence that the gut microbiota plays a key pathogenic role in this model.

## Discussion

Recently, the concept of the gut-kidney axis has attracted attention as an organ connection with a bidirectional link.^20,21^ Gut dysbiosis, defined as abnormalities in the composition and/or quantity of the intestinal microbiota, is caused by various factors, such as metabolic acidosis and a uremic state, oral medications, constipation, and nutritional disorders in chronic kidney disease (CKD). Conversely, dysbiosis affects CKD via uremic toxins, inflammatory responses, and immune mechanisms. Among these factors, uremic toxins, such as IS and p-cresyl sulfate, are fully produced by intestinal bacteria from dietary components and are involved in the progression of CKD.^22^ Of note, IS was reported to induce a proinflammatory phenotype in mouse podocytes, perturb the actin cytoskeleton, decrease the expression of podocyte-specific genes, and reduce cell viability.^15^

Regarding the gut-kidney axis in disorders other than CKD, we previously reported a relationship between gut dysbiosis and childhood INS.^3,6,7^ These studies have shown that the gut microbiota in relapsing INS children has reduced alpha diversity compared with that of healthy controls, as well as gut dysbiosis characterized by a decrease in SCFA, in particular, butyrate-producing bacteria. Since our first report on dysbiosis in children with INS,^3^ a series of similar reports have disclosed dysbiosis characterized by a smaller proportion of beneficial bacteria, such as SCFA-producing bacteria, and an increased abundance of harmful bacteria in patients with INS. Some studies reported that initial therapy with steroids increased SCFA-producing gut microbiota in INS children,^4^ and others postulated that patients with relapsing INS had lower proportions of *Blautia*, *Dialister*, and butyric acid-producing bacteria at onset, and higher proportions of *Oscillibacter*, *Anaerotruncus*, and *UBA1819* than patients without relapse.^9^ Similarly, in adults with INS, gut dysbiosis characterized by a decreased proportion of butyric acid-producing bacteria, such as *Prevotella*, and an increased abundance of harmful bacteria, such as *Escherichia-Shigella*, has been reported.^5,8^ However, the causal relationship between gut dysbiosis and the onset/relapse of INS has not been completely defined.

Taken together, we wondered whether a decrease in beneficial bacteria or an increase in harmful bacteria would have a more critical role in the appearance of proteinuria in INS. Therefore, we designed this study to elucidate the effect of gut dysbiosis on proteinuria in MCNS. If a decrease in the relative abundance of beneficial bacteria contributes to the appearance of proteinuria, depletion of the gut microbiota by antibiotics should exacerbate proteinuria in PAN rats, whereas if an increase in the relative abundance of harmful bacteria contributes to the appearance of proteinuria, depletion of the gut microbiota by antibiotics should alleviate proteinuria. To this end, we compared the urinary levels of protein in conjunction with renal histology and the urinary levels of IS and 8-OHdG between PAN rats treated with and without antibiotics. As a result, we confirmed that administering a combination of antibiotics to deplete gut microbiota in PAN rats had an anti-proteinuric effect. In addition, the administration of antibiotics before PAN injection suppressed the effacement of podocyte foot processes (assessed by electron microscopy) in PAN rats, similar to MCNS in humans. We also confirmed that the administration of antibiotics to PAN rats significantly decreased the levels of urinary IS and 8-OHdG. Additionally, our analysis of gut microbiota in PAN rats treated with antibiotics revealed a decrease in alpha-diversity and the abundance of bacteria that produce indole from tryptophan,^23^ which is metabolized into IS, compared with PAN rats untreated with antibiotics, in agreement with the decreased levels of urinary IS in the antibiotic-treated group.

We further demonstrated that when IS was administered once to rats under conditions where indole-producing bacteria were reduced by administering a combination of antibiotics, their urinary protein levels were markedly increased, suggesting that gut microbiota-derived IS is, at least in part, involved in podocyte injury and the appearance of proteinuria in PAN rats. To provide definitive evidence for this causal relationship, we performed an FMT experiment showing that the profound anti-proteinuric effect of antibiotics was completely abolished when the gut microbiota was restored by transplanting fecal matter from healthy, untreated donors (Fig. 6). This finding causally links the presence of the gut microbiota to the pathogenesis of proteinuria in this PAN-induced nephrosis model. Furthermore, it strongly supports our hypothesis that the protective effects of antibiotics are not related to a direct action of the drugs on the kidney, but are mediated through the depletion of pathogenic components within the gut microbiota. Indeed, recent studies of mice reported that tryptophanase activity possessed by the *Bacteroides* genus, a major bacterial genus in the human intestine, is important for indole metabolism and that the amount of IS in the body is regulated by controlling the abundance of *Bacteroides*.^18^ In agreement, a decrease in the *Bacteroides spp*. was observed in the present study after antibiotic administration, which appeared to be involved in the reduction of urinary IS.

From these findings, we speculated that administering antibiotics 10 days prior to administering PAN to rats would reduce the amount of indole produced by eradicating taxa, such as genus *Alistipes*, genus *Desulfovibrio*, and *Bacteroides spp*. involved in the metabolism of tryptophan in the gut microbiota.^18,19^ Tryptophan, an essential amino acid for humans, is metabolized into indole by the gut microbiota, and indole-3-acetic acid is produced as an intermediate product in the indole metabolic pathway of the gut microbiota.^24^ Although some metabolites produced by the indole metabolic pathway in the gut microbiota have beneficial effects, IS, produced by metabolizing indole itself in the host’s liver, induces oxidative stress, promotes tissue fibrosis, and induces inflammation.^25^ In addition, it was postulated that the aryl hydrocarbon receptor, an IS receptor present in podocytes, leads to the effacement of podocyte foot processes, cytoplasmic vacuoles, or local changes in podocin and synaptopodin.^15^ Considering the possible contribution of oxidative stress to the development of proteinuria in INS,^26–28^ we hypothesized that a decrease in urinary IS and the parallel reduction in urinary 8-OHdG levels related to antibiotic administration in PAN rats would have an anti-proteinuric effect after PAN injection in this study.

This study had some limitations. First, it used rats, and it is unclear whether administering antibiotics to humans in the same way will exert an anti-proteinuric effect on MCNS. Second, applying the results of the present study to clinical practice by the administration of the cocktail of antibiotics to patients with MCNS to suppress its relapse appears to be visionary, as the administration of antibiotics to humans usually causes depletion of the gut microbiota.^29^ In general, although the gut microbiota includes bacteria that are not beneficial to humans, such as indole-producing bacteria, it also includes beneficial bacteria that produce SCFA, such as butyrate. Therefore, when administering antibiotics to patients with MCNS, it is necessary to investigate the type and dosage of drugs that exert an anti-proteinuric effect without reducing beneficial bacteria. Alternatively, an oral spherical activated carbon, AST-120 (Kremezin) that adsorbs uremic toxin precursors such as indole and p-cresol produced in the intestinal tract by intestinal microbiota and promotes their excretion,^30^ is expected to be effective in children with MCNS. This agent is already covered by health insurance in Japan to treat CKD by improving uremic symptoms and delaying the introduction of dialysis.

In conclusion, we report that the administration of a combination of oral antibiotics for 10 days to PAN rats exerted an anti-proteinuric effect associated with a decrease in IS, a gut-microbiota-derived uremic toxin, in parallel with the reduction of urinary 8-OHdG, a marker of oxidative stress. If we can determine the appropriate types and dosages of antibiotics to eradicate indole-producing bacteria exclusively or confirm the efficacy of spherical charcoal adsorbents such as AST-120 to prevent the development of proteinuria in human MCNS, it is expected that the total amount of adrenocortical steroid drugs, which are the first-line drug for MCNS, can be reduced.

## Supplementary information


Supplementary information


## Data Availability

The datasets generated during and/or analyzed during the current study are available from the corresponding author on reasonable request.
