# Supplementary material for: Depletion of gut microbiota alleviates proteinuria in puromycin aminonucleoside-induced nephrosis in rats
Source: Pediatr Res. 2025 Dec 15;100(1):497–505. doi: 10.1038/s41390-025-04668-9 (PMC13524797; doi:10.1038/s41390-025-04668-9)
Supplement: Supplementary file 1 [file 41390_2025_4668_MOESM1_ESM.pdf]

Figure S1

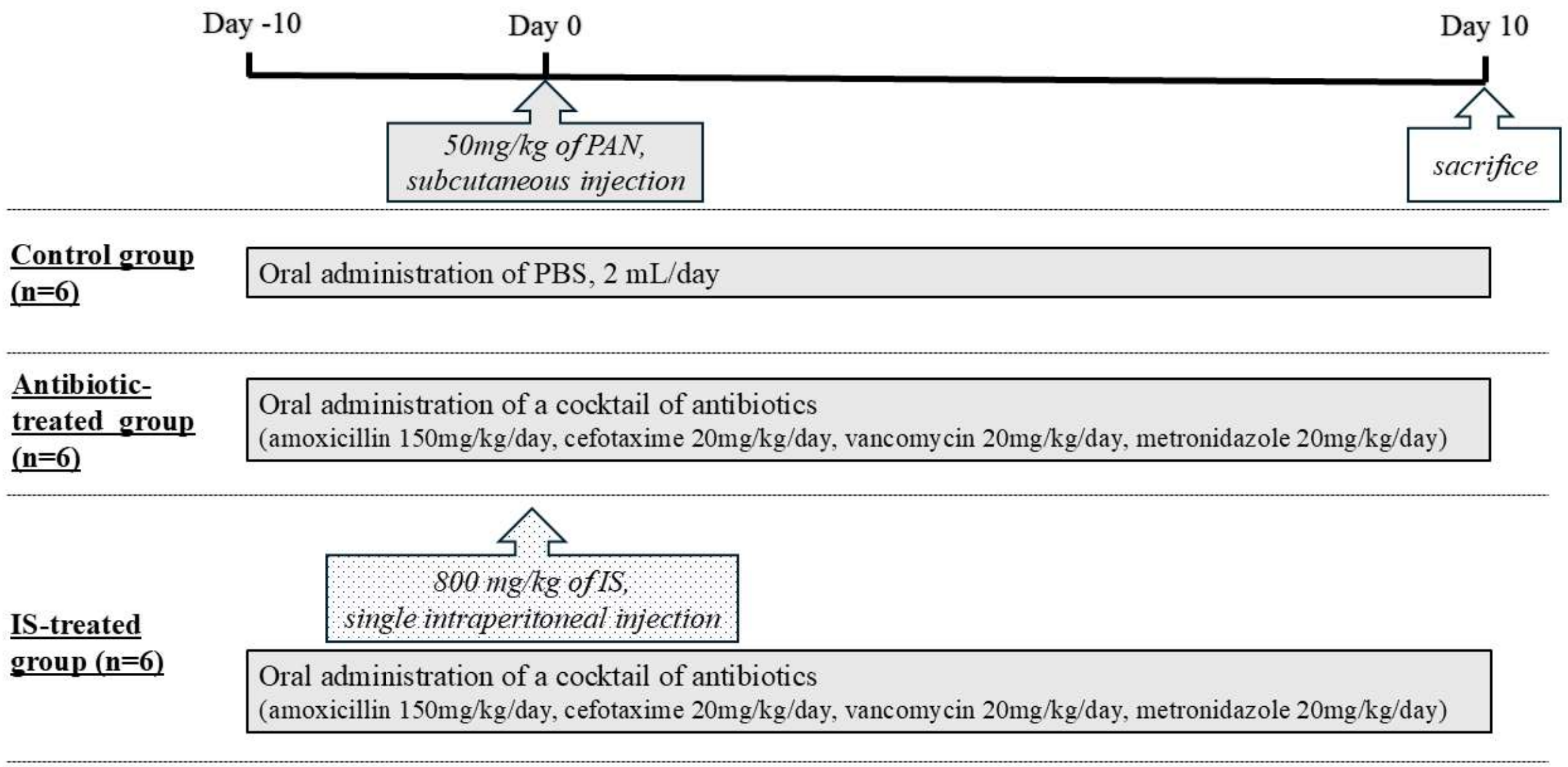

Fig. S1. Schematic diagram of the experimental protocol for the initial study groups.

This timeline illustrates the treatment schedule for the three primary groups. Control group (n=6): rats received the daily oral administration of PBS for 10 days, followed by a single subcutaneous injection of PAN (50 mg/kg) on day 0. Antibiotic-treated group (n=6): rats received a daily oral cocktail of amoxicillin, cefotaxime, vancomycin, and metronidazole for 10 days, followed by a single subcutaneous injection of PAN on day 0. IS-treated group (n=6): rats received the same 10-day antibiotic treatment, followed by a single subcutaneous injection of PAN and a single intraperitoneal injection of IS (800 mg/kg) on day 0. All animals were monitored for 10 days post-PAN injection before sacrifice.

PAN, puromycin aminonucleoside; PBS, phosphate-buffered saline; IS, indoxyl sulfate.

Figure S2

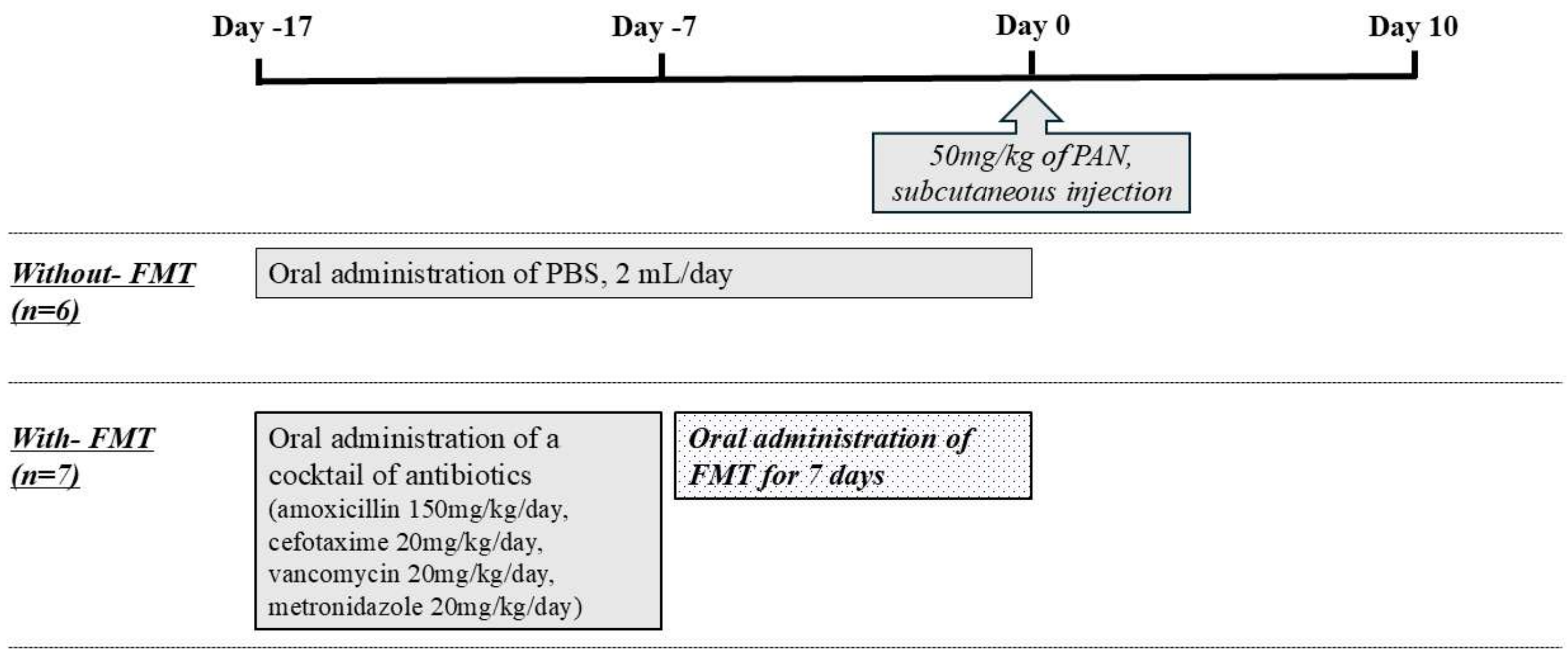

Fig. S2. Schematic diagram of the experimental protocol for the FMT study.

This timeline illustrates the schedule for the FMT experiment. The with-FMT group (n=7): rats were pre-treated with an oral antibiotic cocktail for 10 days (from day −10 to day 0) to deplete the native gut microbiota. This was followed by the daily oral administration of FMT for 7 days. The without-FMT group (n=6): the control group received oral PBS during the same period. Upon completion of the FMT or PBS administration, all rats received a single subcutaneous injection of PAN (50 mg/kg) on day 0 and were monitored for 10 days.

FMT, fecal microbiota transplantation; PAN, puromycin aminonucleoside; PBS, phosphate-buffered saline; OA, oral administration.
